# The effect of posture on the age dependence of neurovascular coupling

**DOI:** 10.14814/phy2.70031

**Published:** 2024-09-01

**Authors:** James D. Ball, Aaron Davies, Dewakar Gurung, Alex Mankoo, Ronney Panerai, Jatinder S. Minhas, Thompson Robinson, Lucy Beishon

**Affiliations:** ^1^ Department of Cardiovascular Sciences University of Leicester Leicester UK; ^2^ NIHR Leicester Biomedical Research Centre, British Heart Foundation Cardiovascular Research Centre Glenfield Hospital Leicester UK

**Keywords:** age, attention stimulation, neurovascular coupling, posture, transcranial Doppler ultrasonography, visuospatial stimulation

## Abstract

Previous studies report contradicting age‐related neurovascular coupling (NVC). Few studies assess postural effects, but less investigate relationships between age and NVC within different postures. Therefore, this study investigated the effect of age on NVC in different postures with varying cognitive stimuli. Beat‐to‐beat blood pressure, heart rate and end‐tidal carbon dioxide were assessed alongside middle and posterior cerebral artery velocities (MCAv and PCAv, respectively) using transcranial Doppler ultrasonography in 78 participants (31 young‐, 23 middle‐ and 24 older‐aged) with visuospatial (VST) and attention tasks (AT) in various postures at two timepoints (T2 and T3). Between‐group significance testing utilized one‐way analysis‐of‐variance (ANOVA) (Tukey post‐hoc). Mixed three‐way/one‐way ANOVAs explored task, posture, and age interactions. Significant effects of posture on NVC were driven by a 3.8% increase from seated to supine. For AT, mean supine %MCAv increase was greatest in younger (5.44%) versus middle (0.12%) and older‐age (0.09%) at T3 (*p* = 0.005). For VST, mean supine %PCAv increase was greatest at T2 and T3 in middle (10.99%/10.12%) and older‐age (17.36%/17.26%) versus younger (9.44%/8.89%) (*p* = 0.004/*p* = 0.002). We identified significant age‐related NVC effects with VST‐induced hyperactivation. This may reflect age‐related compensatory processes in supine. Further work is required, using complex stimuli while standing/walking, examining NVC, aging and falls.

## 1 INTRODUCTION

Cognitive stimulation increases the cerebral demand for oxygen and nutrients due to increased neuronal activation and processing, which is metabolically demanding (Salgado et al., 2016; Palacios‐Filardo & Mellor, 2019; Mather, 2020). This alters nutrient and blood flow distribution within the local cerebrovasculature to meet these demands (Holroyd, 2016; Oyarzabal & Marin‐Valencia, 2019). This coupling between cerebral blood flow (CBF) to meet the metabolic demands of neuronal activity as a result of increased cognitive workload is known as neurovascular coupling (NVC) or functional hyperaemia.

NVC is mediated structurally by the neurovascular unit (NVU) and has been used as a marker to assess cognitive health and to develop mechanistic insights into neurodegenerative disorders including Alzheimer's disease (AD), Parkinson's disease (PD), and amyotrophic lateral sclerosis (ALS) (Attwell et al., 2010; Phillips et al., 2016; Yu et al., 2020). The impact of age on NVC has been frequently investigated, but often with contradictory neutral, increased, or reduced NVC activation (Csipo et al., 2019; Stefanidis et al., 2019; Beishon et al., 2021; Csipo et al., 2021). In previous work, we identified increased NVC responses in older compared to younger adults when presented with cognitive stimuli (Beishon et al., 2019). This may be due to compensatory processes in older adults to maintain their cognitive performance (Lin et al., 2010).

Vascular aging is a gradual phenomenon which increases the risk of neurodegenerative diseases, such as dementia, as a result of functional and structural changes to the vascular system, such as arterial stiffening and dilation (Charlton et al., 2022; Vlachopoulos et al., 2011; Ungvari et al., 2018; Huo et al., 2018; Vatner et al., 2021). This occurs via endothelial alterations and blood–brain barrier malfunction which weakens intracellular protective mechanisms and induces chronic hypoperfusion (Akinyemi et al., 2013). Furthermore, a recent review established an association between accelerated vascular aging and premature mortality. Thus, markers of early vascular aging may enable earlier detection and treatment (Hamczyk et al., 2020).

Transcranial Doppler (TCD) uses low‐frequency (≤2 MHz) ultrasound waves to capture dynamic real‐time measurement of cerebral blood velocity (CBv) as an indirect measure of NVC in response to cognitive stimulation (Sliwka et al., 1995). Typically, insonation occurs through the temporal window, enabling assessments of CBF within the anterior, middle, and posterior cerebral arteries (ACA, MCA, and PCA) (Basri et al., 2021). TCD provides excellent temporal, but relatively low spatial, resolution compared to other neuroimaging modalities and cannot localize neuronal activation to specific brain regions (Csipo et al., 2021). Despite this limitation, TCD has good acceptability and tolerability to patients—especially those with cognitive or mobility problems, which become more likely in older age groups (D'Andrea et al., 2016). Unlike other imaging modalities (e.g., functional magnetic resonance imaging (fMRI), functional near‐infrared spectroscopy (fNIRS)), TCD can assess CBv as a real‐time proxy for CBF in participants in dynamic states, with minimal movement artifact (Naqvi et al., 2013).

In some countries, up to 27% of older adults experience falls (Kalula et al., 2016). Falls are particularly prevalent in those with hypertension, which is commonly associated with orthostatic hypotension and both are particularly prevalent in older people (Abu Bakar et al., 2021; Saedon et al., 2020). Orthostatic hypotension negatively affects cognitive function and increases falls risk, and it is also well documented that differing postures can induce varying blood pressure (BP). For example, reduced BP is present in standing posture compared to seated and supine, especially with increasing age (Eşer et al., 2007; Hofsten et al., 1999; Strumia et al., 2023). The possible mechanism linking falls, cognition, and BP could be a reduced ability for dual tasking (e.g., cognitive processing while walking), which may be due to a reduction in NVC processing during postural changes. Therefore, we hypothesize this effect to be due to it becoming harder to mount sufficient NVC responses in upright postures with age (Eşer et al., 2007).However, few studies have assessed the effects of posture on NVC, with mixed results (Stewart et al., 2015; Garrett et al., 2017; Jor'dan et al., 2018), but even fewer have directly investigated the relationship between age and NVC under different postures (Azevedo et al., 2007; Huo et al., 2018). Therefore, the purpose of this study was to investigate the effect of age on TCD‐measured NVC in three different postures (supine, seated, and standing). We hypothesized increasing age to be associated with reduced NVC activity within more upright postures, particularly amongst older adults.

## 2 METHODS

### 2.1 Study participants

Seventy‐eight healthy participants (41 female, median age 49.5 years [interquartile range (IQR): 23–62 years/IQR = 39 years]) were recruited between October 2021 and April 2022 via the University of Leicester, Join Dementia Research and community groups. Inclusion criteria were healthy adults aged over 18 years, with good understanding of verbal and written English, who were able to perform the study requirements. Healthy adults with well‐managed and stable co‐morbidities, such as hypertension, were accepted. Exclusion criteria were those under 18 years of age, unable to comply with the study, pregnancy or planning pregnancy, lactating, mild cognitive impairment, dementia or neurological degenerative disorders, cardiac diseases (such as severe carotid artery stenosis, heart failure), and severe respiratory disease. Ethical approval (ref: 31663‐ lb330‐ls: cardiovascularsciences) and full written informed consent from each participant were gained before commencement, in accordance with the declaration of Helsinki.

### 2.2 Data collection

Data were collected at the Cerebral Haemodynamics in Aging and Stroke Medicine (CHiASM) research laboratory at the Leicester Royal Infirmary—a temperature controlled (20–24°C) environment with minimal distractions. Participants refrained from alcohol (Balogh et al., 2022), heavy meals (Lavi et al., 2009), and caffeine (Lester et al., 2024) for 4 h and avoided strenuous exercise for 12 h preceding study commencement (Talbot et al., 2023). We followed published guidelines for dCA assessment (Panerai et al., 2023) in the absence of guidelines for NVC. Hand and hemisphere dominance was assessed via Edinburgh Handedness Inventory (Oldfield, 1971). Continuous measurements of the dominant MCA velocity (MCAv), and non‐dominant PCA velocity (PCAv) were made through insonation of the temporal window using 2 MHz TCD probes (DWL Doppler Box QL 2.10.1.2 Software, Singen, Germany) secured with a headframe. Alongside this, continuous, beat‐to‐beat BP was monitored using a non‐invasive finger arterial volume clamping system (Fiometer, Finapres Medical Systems, Amsterdam) on the non‐dominant middle finger, three‐lead electrocardiogram (ECG) assessed heart rate (HR), and end‐tidal carbon dioxide (CO_2_) (EtCO_2_) was assessed via capnograph (Capnocheck Plus oximeter; Smiths Medical, Ashford, UK). Task and rest commencements and terminations were harmonized using a manual marker. Recording rate for all data was 500 samples/s acquired via PHYSIDAS data acquisition software (Department of Medical Physics, University Hospitals of Leicester).

Assessments were conducted in three postures (supine, seated and standing) to mimic everyday situations. Firstly, participants were in a supine position, lying on a couch. Then, participants were seated in a chair, and finally participants stood. Initially, 5 min baseline recording was taken in the supine position, and participants were instructed to close their eyes for the final minute. Following this, participants underwent a visuospatial task (VST) (a dot counting task) lasting 1 min, a one‐min rest period (inactivity) to allow CBv to return to baseline, and then a one‐min attention task (AT) (serial subtraction), with a final minute rest completing the assessment. The participant was then transferred from supine posture to seated posture. The participant was seated resting for 1 min with eyes closed before completing the VST and AT with identical timings (1 min rest in between tasks and after AT). The participant was then transferred to a standing position where the same tasks, rests and durations were completed. Participants for the standing section of the protocol were initially seated for 1 min rest, then stood for 1 min rest, then began the VST and AT, in order to visualize the transition from seated to standing postures. Between the supine and seated conditions, participants also underwent hypo‐ and hypercapnic assessments not included in this analysis. Five‐min washout periods were used between postural conditions. At the end of the haemodynamic assessment, participants underwent a full Addenbrooke's cognitive examination‐III (ACE‐III) to assess global cognitive function (Hsieh et al., 2013).

### 2.3 Visuospatial and attention tasks

We used two paradigms (VST and AT) to evoke CBv responses which we have demonstrated in healthy populations previously (Beishon et al., 2020; Beishon, Panerai, et al., 2018; Beishon, Williams, et al., 2018). These tasks are derived from the ACE‐III which is a global measure of cognitive function used clinically in the process of diagnosing dementia (Bruno & Schurmann Vignaga, 2019; Zarrella et al., 2023). MCAv is assessed in response to the AT and PCAv in response to the VST to match the blood supply to the area of cortical processing, where the MCA supplies lateral brain regions (attention and working memory processing) while PCA supplies posterior basomedial regions (visuospatial processing) (Bleton et al., 2016).

The visuospatial paradigm lasted 1 min and involved participants counting the numbers of dots appearing on a screen silently (Bruckert et al., 2021). The dots were presented in a new formation and quantity every 4 s, equating to 15 random arrangements per 1 min stimulation period. Stimuli were placed close to the participants face (20 cm) and tested with participants prior to the measurements commencing.

The attention paradigm lasted 1 min and involved participants counting backwards from 200 in multiples of six—silently. To avoid task habituation, the number subtracted for the AT (200–5, 6, and 7) was adjusted between conditions, and the presentation (number, order, arrangement) of dots to count was randomized for the VST. In a previous study, we did not find any difference in NVC responses to changing the level of task complexity (Intharakham et al., 2022).

### 2.4 Data preparation and statistical analysis

Data were analyzed offline using software developed by this group previously (Panerai et al., 2012; Panerai et al., 2005). During this process, data were visually examined and rejected if not deemed of sufficient quality for inclusion. Data spikes due to external noise were removed via linear interpolation or median filtering where appropriate and necessary. All remaining data then underwent a zero‐phase Butterworth filter (<20 Hz). Beat‐to‐beat BP, HR and CBv were calibrated with three‐lead ECG readings. Finometer readings were calibrated using brachial BP readings prior to each posture reading. Following pre‐processing, data were normalized to 20s baseline immediately preceding task commencement to determine peak percentage changes at two timeframes (T2 and T3) within each one‐minute stimulation period. T2 signifies initial responses (5–10s after task commencement) while T3 denotes sustained responses (10–20s after task commencement). Rather than considering the complete response using area under the curve (AUC), using two timepoints enables delineation between difference response phases which may be more centrally or more sympathetically mediated (T2 and T3, respectively).

Study participants were separated into three age categories: younger aged <31 years (*n* = 31), middle aged 31–60 years (*n* = 23), and older aged >60 years (*n* = 24). Age was tested as a continuous and grouped variable, producing no analytical differences. CBv was tested as percentage change and absolute values. Unless otherwise stated, data reporting has the format mean (SD), with significance testing by one‐way analysis of variance (ANOVA), with post‐hoc testing by Tukey. A mixed three‐way ANOVA was implemented to detect interactions between task (within subjects), posture (within subjects) and age group (between subjects). One‐way ANOVAs (Tukey post‐hoc testing) were implemented to further explore these findings in the instance of significant effects of interactions. Multiple linear regression was performed where there were significant differences in peripheral parameters, to correct for any potential confounding effect on CBv. Statistical analysis and graph production were completed with IBM SPSS software (v28) (IBM, New York, USA) and GraphPad Prism (v9) (GraphPad, San Diego, USA) for Windows. Statistical significance for all analyses was set at *p* < 0.05.

## 3 RESULTS

### 3.1 Demographics

Of 78 enrolled participants, 41 (52.6%) were female, and median age = 49.5 years [IQR: 23–62 years] (Table 1). There was a significant difference in inter‐group median age (younger = 23.0 [22.0–23.5], middle = 51.0 [47.0–56.0], older = 69.0 [65.5–72.25], *p* < 0.001). 85.9% of participants were right‐hand dominant (younger = 83.9%, middle = 82.6%, older = 91.7%, *p* = 0.06). The majority of participants were Caucasian (75.6%), the proportion of which differed between groups (51.6% younger, 82.6% middle and 100% older, *p* = 0.005). Two participants were excluded from the final analyses owing to unsatisfactory data quality, namely non‐physiological noise too great for linear interpolation.

**TABLE 1:** Baseline participant demographics by age categories.

Characteristic	All	<31 years old (younger)	31–60 years old (middle)	>60 years old (older)	*p*‐value
No. of participants (%)	78	31 (39.7)	23 (29.5)	24 (30.8)	–
Age, years	49.5 [23.0–62.0/39.0]	23.0 [22.0–23.5/1.5]	51.0 [47.0–56.0/9.0]	69.0 [65.5–72.25/6.75]	**<0.001**
Female sex, *n* (%)	41 (52.6)	16 (51.6)	15 (65.2)	10 (41.7)	0.27
Ethnicity *n*, (%)					
Caucasian	59 (75.6)	16 (51.6)	19 (82.6)	24 (100)	**0.005**
Asian (Southeast/Middle East)	19 (24.4)	15 (48.4)	4 (17.4)	0 (0.0)	
Black (African/American)	0 (0.0)	0 (0.0)	0 (0.0)	0 (0.0)	
BMI, kg/m^2^	25.5 (3.81)	23.3 (2.73)	27.2 (4.26)	26.7 (3.24)	**< 0.001**
Mean Systolic BP, mmHg	134 (15.9)	126 (11.7)	132 (13.8)	146 (15.5)	**< 0.001**
Mean Diastolic BP, mmHg	75.3 (9.34)	71.4 (9.27)	77.2 (8.74)	78.6 (8.45)	**0.008**
HR, bpm	69.9 (11.8)	71.1 (10.9)	66.9 (9.5)	70.8 (13.8)	0.45
Handedness, *n* (%) RHD	67 (85.9)	26 (83.9)	19 (82.6)	22 (91.7)	0.62
Hypertension, *n* (%)	14 (17.9)	0.0 (0)	3 (13.0)	11 (45.8)	**<0.001**
High cholesterol, *n* (%)	7 (9.0)	0.0 (0)	0.0 (0)	7 (29.2)	**<0.001**
Depression, *n* (%)	5 (6.4)	2 (6.5)	2 (8.7)	1 (4.2)	0.82
Diabetes Mellitus, *n* (%)	4 (5.1)	1 (3.2)	0.0 (0)	3 (12.5)	0.13

*Note*: Data are provided as mean (± SD), or number/percentage and [IQR]. Significance testing performed using one‐way ANOVA, and Chi‐square and Kruskal–Wallis tests. Significance = *p* < 0.05 (bold).

Abbreviations: BMI, body mass index; BP, blood pressure; HR, heart rate; RHD, right hand dominance.

### 3.2 Baseline readings

Mean MCAv and PCAv differed in younger, middle, and older age groups with MCAv and PCAv greatest in the younger group and decreased with increasing age (Table 2).

**TABLE 2:** Baseline CBv (normocapnia) data recorded in supine, seated and standing conditions before task activation.

Posture	Characteristic	<31 years old (younger)	31–60 years old (middle)	>60 years old (older)	*p*‐value
Supine	No. of participants, *n* (%)	31 (39.7)	23 (29.5)	24 (30.8)	–
	Dominant MCAv (cm/s)	70.5 (13.9)	65.7 (18.9)	57.0 (11.4)	0.050
	Non‐Dominant PCAv (cm/s)	44.4 (17.4)	37.9 (13.8)	35.2 (10.7)	0.060
	MAP (mmHg)	86.3 (10.8)	97.5 (13.2)	99.5 (11.4)	**<0.001** ^a^
	HR (bpm)	65.5 (8.8)	64.9 (8.6)	64.8 (9.8)	0.952
	EtCO_2_ (mmHg)	36.1 (3.6)	36.5 (3.6)	32.4 (5.9)	**0.003** ^a^
Seated	No. of participants, *n* (%)	28 (39.4)	20 (28.2)	23 (32.4)	–
	Dominant MCAv (cm/s)	67.4 (13.5)	60.4 (19.7)	50.8 (12.9)	**0.001** ^a^
	Non‐Dominant PCAv (cm/s)	42.1 (15.9)	35.8 (14.1)	34.2 (8.6)	0.090
	MAP (mmHg)	89.0 (11.0)	101.1 (19.5)	97.4 (16.1)	**0.024** ^a^
	HR (bpm)	68.5 (10.6)	64.1 (8.6)	63.3 (8.6)	0.113
	EtCO_2_ (mmHg)	34.9 (3.9)	34.7 (3.8)	31.2 (5.9)	**0.011** ^a^
Standing	No. of participants, *n* (%)	26 (38.8)	18 (26.9)	23 (34.3)	–
	Dominant MCAv (cm/s)	64.6 (12.9)	58.7 (20.2)	48.4 (12.3)	**0.002**
	Non‐Dominant PCAv (cm/s)	41.0 (13.9)	34.7 (14.3)	32.7 (8.4)	0.058
	MAP (mmHg)	94.3 (6.8)	101.2 (13.7)	96.7 (23.8)	0.385
	HR (bpm)	85.1 (12.8)	77.8 (11.1)	70.7 (9.0)	**<0.001** ^a^
	EtCO_2_ (mmHg)	32.9 (4.1)	34.3 (3.4)	30.6 (6.2)	**0.048** ^a^

*Note*: Data are presented as mean (± SD). Significance testing performed using one‐way ANOVA. Significance = *p* < 0.05 (bold)

^a^ Indicates significant difference as per one‐way ANOVA (95% confidence level).

### 3.3 Between age groups

**FIGURE 1:**
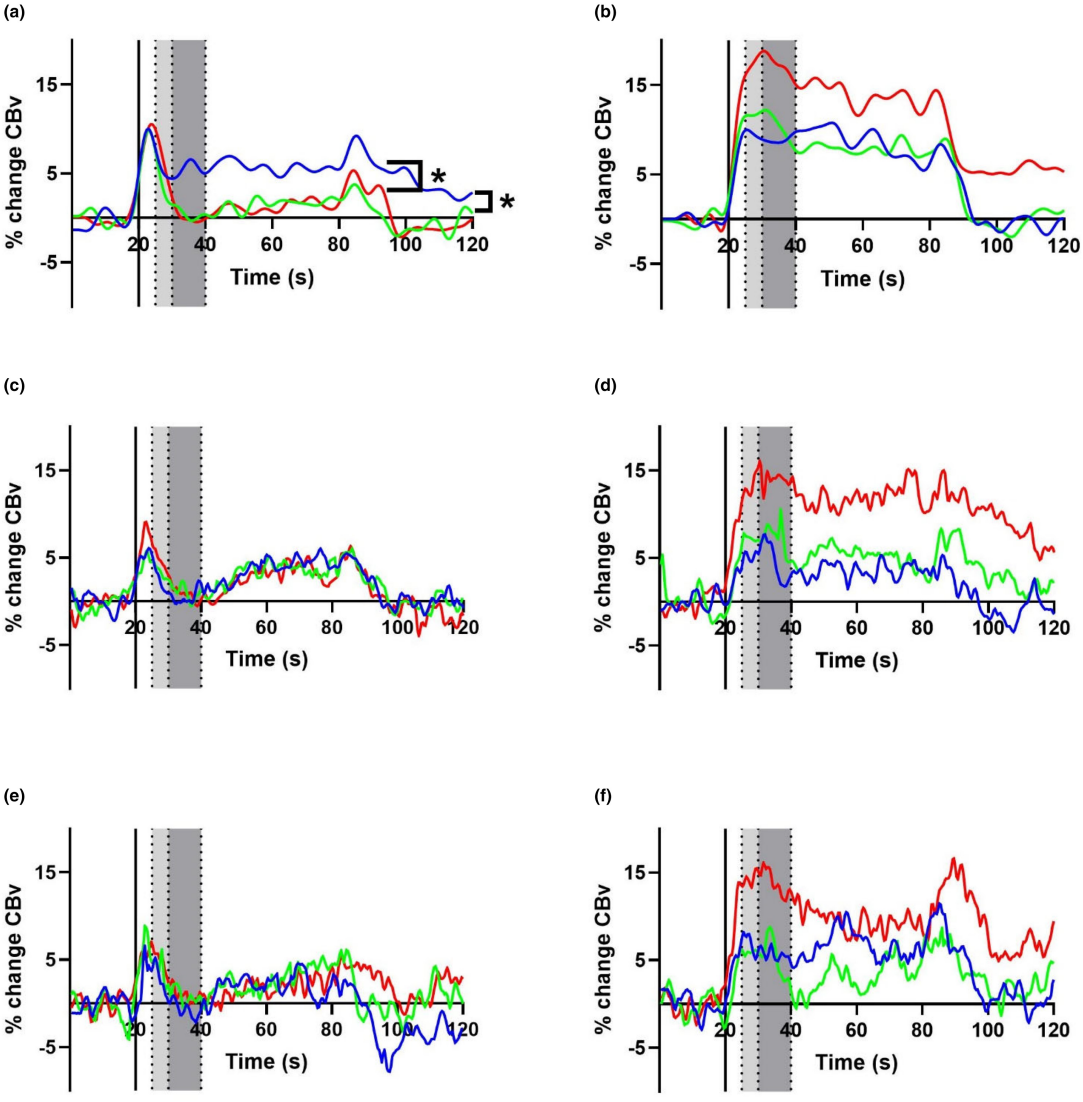
Percentage change in CBv from AT stimulation (left column) (a, c, e) and VST stimulation (right column) (b, d, f) in the MCA and PCA respectively, over the experiment time course. Younger (Blue), middle aged (Green), older (Red). Supine (first row) (a, b) seated (second row) (c, d) and standing (third row) (e, f). Solid vertical like at 20s represents task commencement. Light gray shading signifies T2, and dark gray shading signifies T3. This data shows the population coherent averages of all included participants. ‘*’ indicates significant difference (*p* < 0.05).

During the AT, only supine posture generated elevated %MCAv increase in younger participants over older and middle‐aged participants over the experiment time course, otherwise changes were comparable between age groups (Figure 1). In the VST, the older age group showed greater %PCAv increase in all three postures but most notably in the supine and seated positions.

### 3.4 Attention task

**FIGURE 2:**
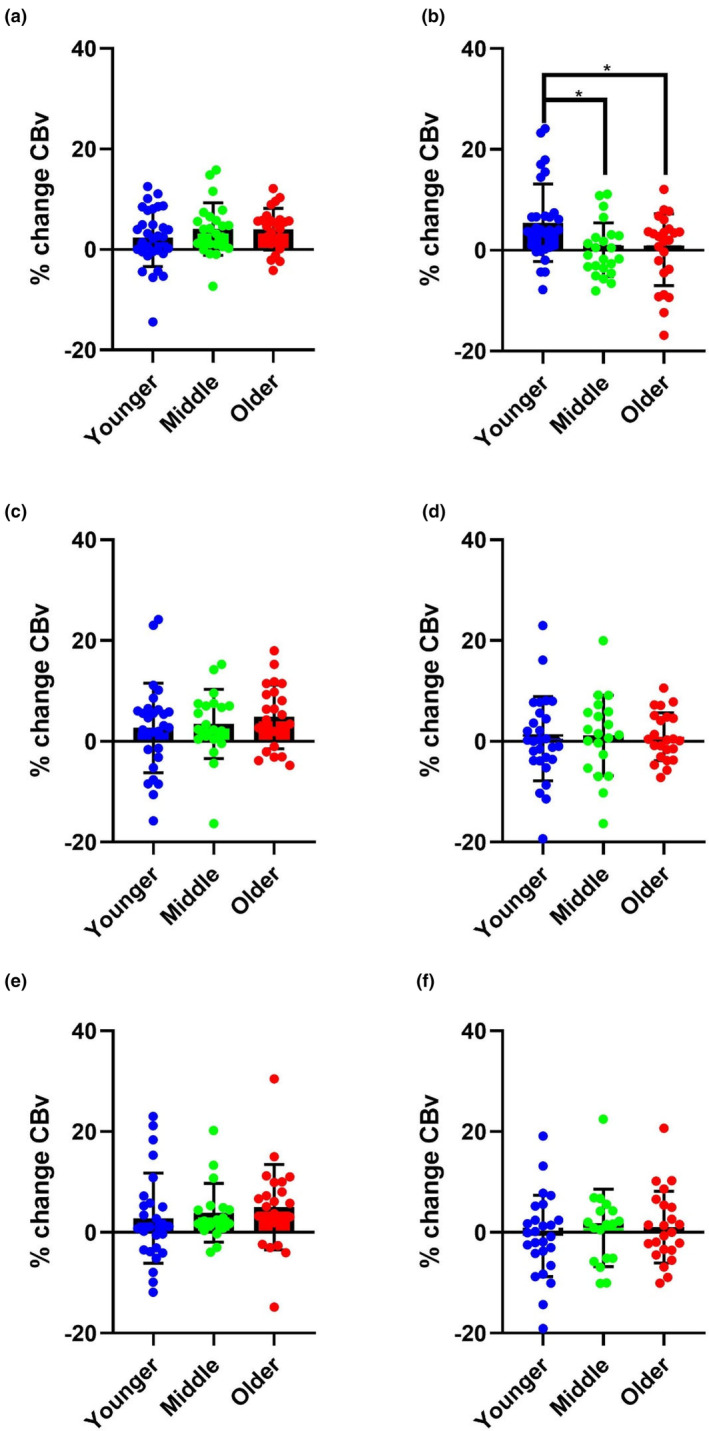
Mean, standard deviation and individual percentage change in MCAv to the AT at T2 (left column) (a, c, e) and T3 (right column) (b, d, f). Younger (Blue), middle aged (Green), older (Red). Supine (first row) (a, b) seated (second row) (c, d) and standing (third row) (e, f). ‘*’ indicates significant difference (*p* < 0.05).

MCAv peak percentage change to the Attention task (AT) in the supine condition differed between younger, middle, and older participants (Figure 2). Mean percentage increase in MCAv to the AT in supine posture was greatest in the younger relative to middle and older age at T3 (5.44% vs. 0.12% and 0.09%, respectively, *p* = 0.005). There were no significant percentage changes in MCAv to the AT T2 (*p* > 0.05). There were no significant percentage changes in MCAv to the AT T2 or T3 in the seated or standing postures (*p* > 0.05).

### 3.5 Visuospatial task

**FIGURE 3:**
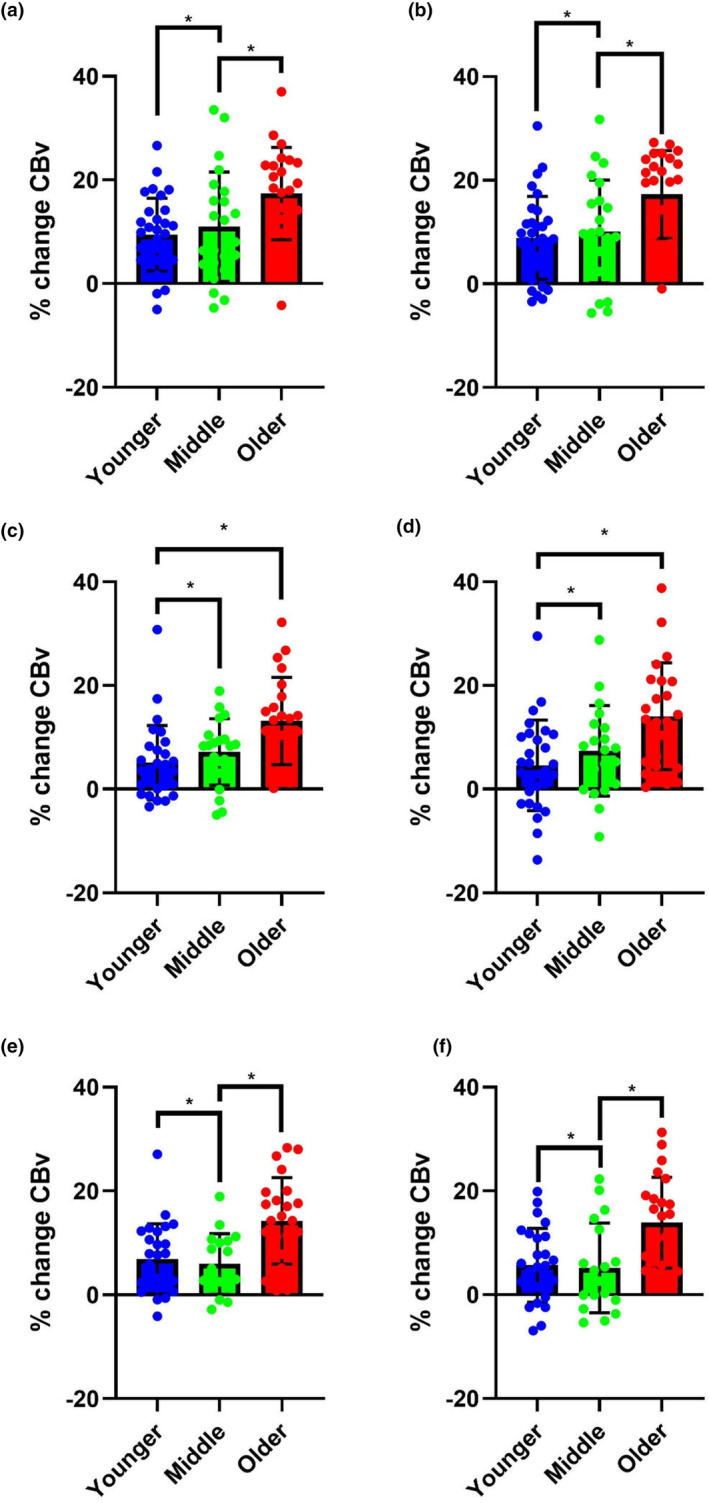
Mean, standard deviation and individual percentage change in PCAv to the VST at T2 (left column) (a, c, e) and T3 (right column) (b, d, f). Younger (Blue), middle aged (Green), older (Red). Supine (first row) (a, b) seated (second row) (c, d) and standing (third row) (e, f). ‘*’ indicates significant difference (*p* < 0.05).

PCAv peak percentage change to the Visuospatial task (VST) in the supine posture differed between younger, middle, and older participants (Figure 3). Mean percentage increase in PCAv to the VST in supine posture was greatest in the middle and older relative to younger age group at T2 (10.99% and 17.36% vs. 9.44%, respectively, *p* = 0.004) and T3 (10.11% and 17.26% vs. 8.89%, respectively, *p* = 0.002). Mean percentage increase in PCAv to the VST in seated posture was greatest in the middle and older relative to younger age group at T2 (7.18% and 13.16% vs. 5.11%, respectively, *p* < 0.001) and T3 (7.39% and 14.09% vs. 4.60%, respectively, *p* = 0.002). Mean percentage increase in PCAv to the VST in standing posture was greatest in the younger and older relative to middle at T2 (6.91% and 14.26% vs. 5.93%, respectively, *p* < 0.001) and T3 (5.66% and 13.88% vs. 5.18%, respectively, *p* < 0.001).

### 3.6 Effect of posture

One‐way ANOVA analysis assessed the effects of posture at time points T2 and T3 and by task (VST and AT). At T2, during the AT, there were no significant differences between any postures. At T2, during the VST, there was a significant increase from seated to supine (3.8%, Standard Error of the Mean (SEM) = 1.40, *p* = 0.021). There were no significant changes in standing posture. At T3, during AT and VST, there were no significant differences between any postures.

### 3.7 Age and posture interaction

Mixed ANOVA of %CBv indicated a significant effect of posture and effect of task at T2 (*p* = 0.003 and *p* < 0.001, respectively) and T3 (*p* = 0.008 and *p* < 0.001, respectively) with significant interaction between age and task at T2 (*p* = 0.018) and T3 (*p* < 0.001).

On post‐hoc testing, at T2 during the AT, there were no significant differences between age groups in any postures. At T3 during the AT, there were significant mean differences between young and middle age %MCAv (5.33%, SEM = 1.90 *p* = 0.017, Cohen's *d* = 0.79) and young and old age (5.35%, SEM = 1.87, *p* = 0.015) in the supine posture, Cohen's *d* = 0.72.

On post‐hoc testing, at T2 during the VST, there were significant differences between young and older %PCAv in the seated (8.05%, SEM = 2.09, *p* = <0.01, Cohen's *d* = 1.04), standing (7.35%, SEM = 2.04, *p* = 0.002, Cohen's *d* = 0.98), and supine (7.92%, SEM = 2.38, *p* = 0.004, Cohen's *d* = 1.00) postures. In all three postures, there were also significant differences between middle and old age (5.98%, SEM = 2.27, *p* = 0.027, Cohen's *d* = 0.79, 8.33%, SEM = 2.24, *p* = 0.001, Cohen's *d* = 1.13 and 6.37%, SEM = 2.55, *p* = 0.039, Cohen's *d* = 0.66, respectively).

On post‐hoc testing, at T3 during the VST, there were significant differences between young and older %PCAv in the seated (9.49%, SD = 2.61, *p* = 0.002, Cohen's *d* = 1.00), standing (8.23%, SD = 2.32, *p* = 0.002, Cohen's *d* = 1.04), and supine (8.38%, SD = 2.38, *p* = 0.002, Cohen's *d* = 1.02) postures. In the standing, and supine postures, there were also significant differences between middle and old age (8.70%, SD = 2.55, *p* = 0.003, Cohen's *d* = 1.00, and 7.15%, SD = 2.55, *p* = 0.018, Cohen's *d* = 0.78, respectively).

### 3.8 Absolute values

Data was explored as %CBv change and absolute CBv change. When absolute values were explored, there was a significant effect of posture (*p* < 0.001), but there was no significant effect of task (*p* = 0.113) and there was no interaction between task and age (*p* = 0.402). One‐way ANOVA analysis assessed the effects of posture at time points T2 and T3 and by task (VST and AT) in absolute CBv values. At T2, during the AT, there were no significant differences between any postures. At T2, during the VST, there was a significant increase from seated to supine (1.77 cm/s, SEM = 0.52 cm/s, *p* = 0.002) and standing to supine (1.69 cm/s, SEM = 0.52 cm/s, *p* = 0.004). At T3, during AT, there were no significant differences between any postures. At T3, during VST, there was a significant increase from seated to supine (1.47 cm/s, SEM = 0.58 cm/s, *p* = 0.034) and standing to supine (1.61 cm/s, SEM = 0.59 cm/s, *p* = 0.02).

Within the peripheral parameters assessed (MAP, HR and EtCO2), there were significant differences in response to both the AT and VST (see Supplementary Material Table S1.1/2—Data S1). Following correction for significant differences in peripheral parameters, there were no effects of MAP, HR or EtCO2 and the effect of age remained significant.

Age was explored as a continuous variable throughout but did not change the main results compared with the use of three distinct age groups.

## 4 DISCUSSION

### 4.1 Main findings

There was a significant effect of posture on NVC, with a 3.8% increase from seated to supine postures. This may indicate compensatory processes optimizing NVC and cognitive function under seated and supine conditions, but not standing. There was a significant interaction between age and task. However, we did not identify any interaction between age and posture on NVC across two vascular territories.

### 4.2 Effects of age and posture on NVC

Notably, within the VST, in all three postures, there is marked hyperactivation shown in the older age group PCA (Figure 1). Hyperactivation may be due to compensatory processes in older people who require greater neural resources to maintain comparable cognitive performance levels to younger people or central command inducing CBv fluctuations (Reuter‐Lorenz & Cappell, 2008). However, this hyperactivation may also be impacted by increased CBv requirements to meet similar neural activation to those of younger participants. Arterial stiffening, amplified pulsatility and increased total peripheral resistance may occur in older participants, but declining cognitive function induces hyperactivation as executing the same level of neuronal function requires more energy resources because of a reduction in processing efficiency (Tarumi & Zhang, 2018). Age‐related cognitive decline may also associate with reduced frontal brain region white matter integrity and gray matter volume reductions (Park & Reuter‐Lorenz, 2009).

Several theories may explain the NVC differences associated with aging. The posterior‐to‐anterior shift in aging (PASA) model was presented after cerebral activation scrutiny showed reduced posterior and increased anterior activation with age (Davis et al., 2008). An alternative theory is the hemispheric asymmetry reduction in older adults (HAROLD). HAROLD proposes age‐related prefrontal cortex functional hemispheric lateralisation impairment (Cabeza et al., 2002). Finally, the compensation‐related utilization of neural circuits hypothesis (CRUNCH) suggests extra brain regions outside the contralateral hemisphere are recruited to resolve the greater cognitive load with increasing age (Reuter‐Lorenz & Cappell, 2008). However, further analysis of the HAROLD and CRUNCH hypotheses using younger and older fMRI patterns, and quantitative voxel‐by‐voxel statistical evaluation, concluded HAROLD insufficiently summarizes age‐related cerebral activation changes, with some effects comparable to HAROLD presenting outside the prefrontal cortex (Berlingeri et al., 2013). The analysis concluded the more generalized activation of the CRUNCH hypothesis is more aligned with compensatory age‐related neural activation pattern changes.

One study assessed visual stimulation using identical postures to this study, but without reporting effects of age (Azevedo et al., 2007). The study identified no significant NVC between‐posture differences, which was mirrored by other literature (Camden et al., 2021). As discussed, hyperactivation occurred responding to VST, not AT in older participants. This may be attributable to lower AT cognitive load being insufficient to require compensatory hyperactivation (Csipo et al., 2021).

Another study used a dynamic vessel analyzer (DVA)‐based approach to assess age‐related NVC changes (Lipecz et al., 2019). In keeping with our findings, there were significant age‐related effects upon healthy NVC. Both results can be attributed to sympathetic, parasympathetic, and somatic sensory nerve activation eliciting neurogenic blood vessel innervation control, alongside autonomic regulation (Hotta, 2016; Shiogai et al., 2010). Neurogenic innervation control differs between seated/supine and standing postures by correcting for reduced venous return, reducing BP and inducing aortic arch, and carotid sinus baroreceptor firing reductions. These reductions increase neural outflow, vascular tone, stroke volume and HR, maintaining cerebral perfusion pressure, and increasing NVC (Charkoudian & Rabbitts, 2009).

Azevedo et al., studied mainly younger participants (26.8 (±8.8) years). However, both findings demonstrate a significant effect of posture, but with no interaction with age. Here, the effect of posture was driven by a significant 3.8% increase in CBv from seated to supine, possibly attributable to hydrostatic differences between heart and brain, which occur between supine and seated, but not standing (Azevedo et al., 2007). In addition, there was no comparison assessed here between transitioning from supine to seated and supine straight to standing meaning the seated position may provide a transitionary phase—this requires further analysis. Also, due to reductions in cerebral perfusion pressure, compensatory NVC increases required to maintain cognitive performance, may be too great to sustain while standing, meaning NVC returns to supine levels from those seen in seated (Roerdink et al., 2011). This may cause those dual‐tasking in upright posture (e.g., walking/talking situations reflective of everyday life) to be unable to sufficiently increase NVC to maintain cognitive performance, increasing the risk of falls. This is clinically relevant, given it has been demonstrated that cognitive impairment is associated with increased rates of falling partly due to the role cognition has in gait control (Racey et al., 2021). Compromised NVC while standing may increase fall vulnerability if CBF rises cannot meet additional demands of external cognitive stimulation due to reduced NVC capacity. This may be particularly apparent when walking, especially in older participants (Montero‐Odasso et al., 2012). Also, impaired NVC is associated with reduced gait speed compared to faster walkers, although NVC and walking assessments were separate (Sorond et al., 2011).

### 4.3 Strengths and limitations

This study is the first TCD‐assessed NVC investigation of various postures (supine, seated, and standing) using multiple paradigms across a spectrum of ages and vascular territories allowing unique mechanistic insight into age‐related variations in both parameters. This enabled detailed analysis of parameter impacts on CBv changes both in series and independently. Previous studies analyzed these parameters individually but scarcely together (Azevedo et al., 2007; Camden et al., 2021). One limitation associated with this, however, is the use of supine, seating and standing postures in the same order throughout all experiments due to practical reasons. Randomizing the postures was not possible as participants were stabilized before experiments began, which may have had an impact upon results produced. Future studies addressing the potential influence of randomization would be of interest.

Two major limitations exist regarding TCD: the assumption that insonated vessel diameter remains constant and lack of spatial resolution (although off‐set by excellent temporal resolution) (Ali, 2021). Another limitation is only stationary posture inclusion and lack of age‐related gait change assessments. The lack of NVC compensation while standing requires further investigation into potentially increased fall risk (Montero‐Odasso et al., 2012). Therefore, addition of walking conditions would enable falls risk and NVC, to be further evaluated as functions of age and posture. Another limitation worthy of consideration is significance within the ethnicity of varying age groups, limiting conclusions that can be extracted. We acknowledge the lack of randomization within the study protocol which, with greater sample sizes, may facilitate further between‐group analysis in future studies. We further acknowledge that the signal to noise ratio of the data would have been improved, had more repeats of stimuli been implemented. Single repeats of tasks were employed for practical reasons to reduce overall protocol duration, but future work of a similar nature implementing repeated stimuli within their protocols would be of interest.

### 4.4 Future work

Supine, seated, and standing are only three from a spectrum of available postural positions. Further analysis could incorporate more mobile postures alongside different degrees of tilt between the already‐studied postures to provide more comprehensive representations of NVC with posture. This may relate to acute illness hospital bed position variability with reference to the impact of supine and head‐up tilt positions upon CBF (Lam et al., 2018).

Future research could incorporate other imaging modalities, addressing TCD limitations. Spatial resolution of TCD could be improved by combining with other modalities including fMRI or fNIRS (Chen et al., 2020; Pinti et al., 2020), although, with existing technology, fMRI would not be possible with multiple postures.

Future analysis could also incorporate effects of sex, menopause and hormonal cycle on NVC in different postures. Cerebrovascular reactivity is affected by menstruation stage while the effects of menstruation on NVC are vastly under‐researched (Krejza et al., 2013). Younger females with regular menstrual cycles are subject to lesser BP and elevated systemic vascular reactivity preceding ovulation but impacts on NVC are unclear (Adkisson et al., 2010). This would be ample opportunity to assess menstrual stage in a comparable manner to age and posture.

Future work may assess the impact of increasing task complexity including working memory during upright postures with age to gauge risk of falling as increased cognitive demand induced by increasing task complexity may be associated with compensatory hyperactivation. However, reduced NVC in slow walkers indicates walking and cognitive activity may challenge aging participants (Sorond et al., 2011; Burma et al., 2021).

A final future research area is translation into patient populations. Previous research has assessed significant effects of AD (Tarantini et al., 2017) and stroke (Beishon & Minhas, 2021) on NVC. However, interactions these disorders have with posture are less commonly explored. This research presents unique opportunity to conduct comparable investigations into effects of cognitive disorders on posture‐related NVC.

## 5 CONCLUSION

In summary, there is a significant effect of posture on NVC, where %CBv increases most in supine posture relative to seated, but not standing. However, NVC under different postures is not affected by age. We identified significant age‐related effects on task activation with hyperactivation to VST but not AT. This may reflect compensatory processes occurring to maintain cognitive performance with age. Further work is needed to extend these findings, using more complex stimulation tasks in standing and walking postures to determine relationships between NVC, falls risk and aging.

## AUTHOR CONTRIBUTIONS

**James D. Ball**: Conducted analysis and prepared manuscript. **Aaron Davies**: Collected data and conducted analysis. **Dewakar Gurung**: Collected data and conducted analysis. **Alex Mankoo**: Reviewed manuscript. **Ronney Panerai**: Developed software and reviewed manuscript. **Jatinder S. Minhas**: Reviewed manuscript. **Thompson Robinson**: Reviewed manuscript. **Lucy Beishon**: Methodological development and prepared manuscript.

## FUNDING INFORMATION

JB is a PhD Student funded by the NIHR East Midlands ARC. TGR is an NIHR Senior Investigator. LB is an Academic Clinical Lecturer funded by the NIHR. The views expressed are those of the author(s) and not necessarily those of the NIHR or the Department of Health and Social Care.

## CONFLICT OF INTEREST STATEMENT

None to declare.

## ETHICS STATEMENT

Ethical approval (ref: 31663‐ lb330‐ls:cardiovascularsciences) and full written informed consent from each participant were gained before commencement, in accordance with the declaration of Helsinki.

## Supporting information


Data S1:

## Data Availability

The data used in this study is held in the Cerebral Haemodynamics in Aging and Stroke Medicine (CHiASM) research database at the University of Leicester. Data can be provided upon request to the corresponding author.
