# Supplementary material for: The effect of posture on the age dependence of neurovascular coupling
Source: Physiol Rep. 2024 Sep 1;12(17):e70031. doi: 10.14814/phy2.70031 (PMC11366444; doi:10.14814/phy2.70031)
Supplement: Supplementary file 1 — Data S1: [file PHY2-12-e70031-s001.docx]

| **Age** | **Younger** | | **Middle** | | **Older** | | **Between-age group significance P-Values** | |
| --- | --- | --- | --- | --- | --- | --- | --- | --- |
| **Timepoint** | **T2** | **T3** | **T2** | **T3** | **T2** | **T3** | **T2** | **T3** |
| **Seated** | | | | | | | | |
| **CBv1** | 0.6 (3.3) | -0.2 (3.4) | 0.8 (2.3) | -0.3 (2.2) | 1.2 (2.8) | -0.3 (2.7) | 0.77 | 0.98 |
| **CBv2** | 1.6 (6.2) | 0.4 (6.0) | 2.4 (4.2) | 1.0 (4.9) | 2.7 (3.1) | 0.4 (2.6) | 0.72 | 0.92 |
| **MAP** | 1.2 (3.2) | 0.8 (2.7) | 0.1 (4.6) | -0.1 (4.5) | 3.6 (5.3) | 3.2 (3.9) | **0.035*** | **0.014*** |
| **HR** | 2.7 (3.9) | 2.4 (3.8) | 2.5 (2.7) | 2.0 (2.4) | 1.8 (1.7) | 1.6 (2.0) | 0.57 | 0.70 |
| **EtCO2** | 0.2 (0.9) | 0.0 (1.0) | 0.5 (1.6) | 0.7 (1.8) | 0.2 (1.2) | -0.2 (1.4) | 0.61 | 0.14 |
| **Standing** | | | | | | | | |
| **CBv1** | 0.6 (3.9) | -1.3 (4.5) | -0.4 (2.2) | -1.2 (3.2) | 0.3 (2.0) | -1.2 (2.8) | 0.59 | 0.99 |
| **CBv2** | 2.2 (6.2) | -0.4 (5.9) | 2.2 (4.1) | 1.1 (5.2) | 2.3 (3.6) | 0.5 (3.0) | 1.00 | 0.61 |
| **MAP** | -0.2 (2.9) | 0.2 (2.6) | 0.8 (3.5) | 1.1 (3.2) | 1.9 (4.3) | 1.6 (3.7) | 0.15 | 0.32 |
| **HR** | 1.4 (4.7) | 0.9 (5.2) | -0.1 (4.8) | 0.0 (3.3) | 1.6 (4.1) | 0.6 (2.6) | 0.44 | 0.80 |
| **EtCO2** | -0.2 (1.2) | -0.1 (1.2) | -0.5 (1.1) | -0.6 (1.5) | -0.3 (1.2) | -0.5 (1.2) | 0.77 | 0.53 |
| **Supine** | | | | | | | | |
| **CBv1** | 1.7 (2.6) | 1.3 (2.6) | 0.0 (2.1) | -0.9 (2.1) | 1.6 (1.7) | -0.2 (2.6) | **0.019*** | **0.005*** |
| **CBv2** | 3.9 (5.0) | 3.7 (5.6) | 2.7 (2.9) | 0.4 (3.5) | 3.7 (3.6) | 0.0 (4.0) | 0.53 | **0.007*** |
| **MAP** | 1.9 (4.6) | 0.7 (3.3) | 3.8 (4.4) | 2.4 (4.5) | 4.2 (4.1) | 3.0 (4.6) | 0.14 | 0.12 |
| **HR** | 2.2 (4.6) | 3.3 (5.4) | 2.6 (4.1) | 4.1 (3.7) | 2.6 (3.2) | 3.0 (3.7) | 0.93 | 0.68 |
| **EtCO2** | 0.0 (0.9) | 0.1 (1.1) | -0.9 (1.3) | -1.2 (1.8) | -0.4 (1.2) | -0.4 (1.7) | **0.021*** | **0.017*** |

**Table S1.1: Absolute peripheral and cerebral haemodynamic parameter data presented as difference from T1 (task onset) and T2 (25-30 seconds) and T3 (30-40 seconds) for the AT task in different postures and age groups. Data are presented in the format: mean (standard deviation), with between group significance testing by one-way ANOVA. Significance highlighted by * (p<0.05).**

| **Age** | **Younger** | | **Middle** | | **Older** | | **Between-age group significance P-Values** | |
| --- | --- | --- | --- | --- | --- | --- | --- | --- |
| **Timepoint** | **T2** | **T3** | **T2** | **T3** | **T2** | **T3** | **T2** | **T3** |
| **Seated** | | | | | | | | |
| **CBv1** | 1.9 (2.6) | 1.8 (3.4) | 3.0 (2.3) | 2.9 (3.8) | 3.9 (2.2) | 4.1 (2.6) | **0.021*** | 0.07 |
| **CBv2** | 4.7 (4.6) | 3.5 (5.9) | 4.4 (4.6) | 4.2 (4.6) | 3.4 (2.1) | 3.9 (3.7) | 0.55 | 0.88 |
| **MAP** | -0.9 (2.6) | -0.4 (2.8) | -1.0 (3.6) | -0.3 (3.1) | 0.1 (4.1) | 1.4 (5.0) | 0.54 | 0.20 |
| **HR** | 1.1 (3.4) | 1.5 (4.7) | 0.4 (2.6) | 0.7 (2.5) | 0.1 (3.0) | 0.1 (2.4) | 0.48 | 0.39 |
| **EtCO2** | 0.1 (2.3) | -0.3 (1.8) | -0.2 (0.8) | -0.3 (1.1) | -0.3 (1.0) | -0.7 (1.4) | 0.70 | 0.60 |
| **Standing** | | | | | | | | |
| **CBv1** | 2.4 (2.3) | 2.1 (2.9) | 2.2 (2.3) | 1.9 (3.3) | 4.2 (2.8) | 4.0 (2.8) | **0.018*** | **0.045*** |
| **CBv2** | 4.5 (3.6) | 3.4 (3.7) | 3.7 (4.5) | 3.4 (3.3) | 3.0 (2.4) | 3.4 (2.7) | 0.38 | 1.00 |
| **MAP** | -0.1 (3.2) | -0.3 (2.5) | -1.1 (3.5) | -0.5 (2.4) | -0.2 (3.8) | 1.2 (2.3) | 0.66 | 0.06 |
| **HR** | 4.7 (5.7) | 3.4 (4.7) | 1.4 (2.5) | 0.9 (3.0) | 0.7 (2.3) | 0.7 (2.5) | **0.003*** | **0.023*** |
| **EtCO2** | 0.0 (2.0) | -0.6 (3.4) | -0.3 (1.1) | -0.5 (1.3) | -0.3 (1.9) | -0.7 (1.8) | 0.84 | 0.95 |
| **Supine** | | | | | | | | |
| **CBv1** | 3.8 (3.2) | 3.4 (3.9) | 4.4 (4.4) | 4.0 (4.1) | 6.0 (3.9) | 5.8 (3.4) | 0.11 | 0.06 |
| **CBv2** | 6.4 (4.1) | 5.1 (4.1) | 4.5 (4.7) | 4.0 (4.1) | 7.4 (4.2) | 6.3 (3.8) | 0.08 | 0.16 |
| **MAP** | -1.0 (4.2) | -1.3 (3.7) | 0.0 (3.6) | 0.0 (4.3) | 1.7 (5.0) | 3.0 (4.6) | 0.08 | **0.002*** |
| **HR** | 2.3 (4.2) | 3.5 (5.7) | 1.5 (3.9) | 2.8 (4.5) | 2.0 (3.3) | 2.4 (3.6) | 0.77 | 0.67 |
| **EtCO2** | 0.1 (0.8) | 0.3 (0.8) | -0.4 (1.1) | -0.4 (1.0) | -0.1 (1.5) | -0.4 (1.3) | 0.32 | **0.017*** |

**Table S1.2: Absolute peripheral and cerebral haemodynamic parameter data presented as difference from T1 (task onset) and T2 (25-30 seconds) and T3 (30-40 seconds) for the VST task in different postures and age groups. Data are presented in the format: mean (standard deviation), with between group significance testing by one-way ANOVA. Significance highlighted by * (p<0.05).**
